# To see ourselves as others see us

**DOI:** 10.1186/2045-4015-1-2

**Published:** 2012-01-30

**Authors:** Martin McKee

**Affiliations:** 1 London School of Hygiene and Tropical Medicine, Department of Health Services Research and Policy, 15-17 Tavistock Place, London, WC1H 9SH, UK

## Abstract

There is enormous scope to learn lessons from health systems in other countries. The challenge is to learn the right lessons. The Israel Journal of Health Policy Research offers an important new platform where researchers and policy makers from different countries can come together to understand each other and share their experiences.

This is a commentary on http://www.ijhpr.org/content/1/1/1/

## Commentary

"It all depends". And that is the problem. Health policy making is not like civil engineering. Building a bridge across a river in the United States is very much the same as building one in China. Building a health system, as both of those countries are currently realizing, is much more difficult [1,2]. Health systems, like all complex adaptive human systems, are shaped by their environments, their cultures (what the soft systems experts term *Weltanschauung*, or world vision), and by their participants [3,4].

Yet that is only the beginning. Bridges only have to provide a means of getting from A to B. Health systems must do many things, and the importance placed upon them will depend on who you are [5]. As a potential patient, one expects that the system will be there to provide care when it is needed. As someone paying taxes or health insurance contributions, one hopes it will do so in a cost-effective way. As a public health professional, one would hope that it would strive to prevent people becoming ill in the first place. As a health professional, one hopes that it will provide the training, facilities, and technology necessary to do one's job well. As a human rights activist, one hopes that it will treat people fairly and with dignity. As a central banker, one hopes that it will contribute to macroeconomic stability and economic growth (for example, by preventing the need to hoard cash because of fear of catastrophic expenditure or by supporting the biotechnology industry). And as a local politician, one hopes that it will bring employment and infrastructure to one's constituency.

The task of understanding, and hopefully improving these diverse functions, falls to researchers from a very wide range of disciplinary perspectives. They embrace quantitative and qualitative approaches and, increasingly, mixed methods. They draw on the arts, such as history, and the sciences, such as pharmacology and engineering. They use experimental and non-experimental methods, with comparisons across places and over time [6].

At this point the reader could be forgiven for abandoning the quest to understand health policy, instead trusting those who believe they can make some sense to get on and do it. Yet this would be an abdication of responsibility. For too long, we have been doing just this, leaving it to the professional politicians to create policies on the basis of hunches, intuition, anecdote, or, rather too often, ideology (exemplified by the current dismantling of the English National Health Service) [7].

What we need is a place where we, whether researchers, policy advisors, or politicians, can find the diverse information and evidence that we need, in a synthesized form and, crucially, applied to the context we find ourselves in. It is the last requirement that is the challenge, unless, that is, you are an American where you are spoilt for choice with journals such as *Health Affairs, Milbank Quarterly, Health Services Research*, and many others. Yet, all of these journals devote much of their content to something that ceased being an issue for other developed countries over fifty years ago--the question of how to provide health care for all. This is why the *Israel Journal of Health Policy Research *is so important. The Israeli health care system has many of the features of health systems in other high income countries, has achieved many of the same successes, but faces many of the same challenges [8,9]. How do you respond to the challenges of an aging population? How do you meet the needs of an increasingly multi-cultural population? How do you manage the introduction of expensive new technology? As a regular visitor to Israel, I am aware of the remarkable capacity of the Israeli health research community, its willingness to innovate, and its capacity to learn lessons from elsewhere. Yet lesson learning is not straightforward [10]. It demands that we ask not just "will it work?" but "what are the conditions in which it will work?" and "do those conditions exist in my situation?" [11]. Above all, it requires a process of enquiry and reflection, ideally one that involves those from elsewhere who can challenge one's assumptions and ask "why does it have to be so". To quote Robert Burns, "O would some power the gift to give us to see ourselves as others see us." [12]. For those interested in health policy in Israel, whether as participants or observers from afar, this new journal is that gift.

## Competing interests

The author declares that he has no competing interests.

## Author's information

Martin McKee is professor of European Public Health at the London School of Hygiene and Tropical Medicine and Director of Research Policy at the European Observatory on Health Systems and Policies.
